# Tumor-specific CD4^+^ T cells eradicate myeloma cells genetically deficient in MHC class II display

**DOI:** 10.18632/oncotarget.11946

**Published:** 2016-09-10

**Authors:** Anders Tveita, Marte Fauskanger, Bjarne Bogen, Ole Audun Werner Haabeth

**Affiliations:** ^1^ Centre for Immune Regulation, Department of Immunology, University of Oslo and Oslo University Hospital, Oslo, Norway; ^2^ KG Jebsen Centre for Research on Influenza Vaccines, Institute of Immunology, University of Oslo and Oslo University Hospital, Oslo, Norway

**Keywords:** CD4+ T cells, MHC class II, tumor immunology, antigen presentation, anti-tumor immunity

## Abstract

CD4^+^ T cells have been shown to reject tumor cells with no detectable expression of major histocompatibility complex class II (MHC II). However, under certain circumstances, induction of ectopic MHC II expression on tumor cells has been reported.

To confirm that CD4^+^ T cell-mediated anti-tumor immunity can be successful in the complete absence of antigen display on the tumor cells themselves, we eliminated MHC II on tumor cells using CRISPR/Cas9. Our results demonstrate that ablation of the relevant MHC II (I-E^d^) in multiple myeloma cells (MOPC315) does not hinder rejection by tumor-specific CD4^+^ T cells. These findings provide conclusive evidence that CD4^+^ T cells specific for tumor antigens can eliminate malignant cells in the absence of endogenous MHC class II expression on the tumor cells. This occurs through antigen uptake and indirect presentation on tumor-infiltrating macrophages.

## INTRODUCTION

Adoptive transfer of tumor-specific CD4^+^ T cells can mount impressive anti-tumor immune responses in murine tumor models [1–3]. Recent clinical trials also point to a therapeutic benefit of CD4^+^ T cells in humans [4–6]. High-throughput bioinformatics surveys in various tumor models identify a large number of immunogenic MHC class II neoepitopes, and vaccination with such neoepitopes can elicit protective immune responses including epitope spreading [7]. Analysis of human cancer samples revealed a similar accumulation of MHC II neoepitopes [7], and a clinical trial of vaccination using such targets was recently initiated (NCT02035956).

To date, knowledge of the mechanistic basis of anti-tumor CD4^+^ T cell responses is limited. As discussed below, the complex nature of CD4^+^ T cell antigen recognition and effector functions implicates a number of potential effectors. Insight into such processes is therefore of relevance in guiding the development and refinement of such therapy.

It has been suggested that CD4^+^ T cells may be of limited impact in immunotherapy, since most tumor cells lack expression of MHC class II molecules, and thus are not directly recognized by CD4^+^ T cells. However, in several tumor models, CD4^+^ T cells can induce killing of apparently MHC II-negative (MHC II^NEG^) cancer cells [8–12]. This is thought to occur through indirect mechanisms involving activation of M1 macrophages [13] or NK cells [12] within the tumor bed. However, these previous results are not conclusive, since tumor cells under certain circumstances, such as inflammation, may show expression of MHC class II, and thus be subject to direct killing by CD4^+^ T cells.

Expression of MHC II in healthy tissues is under strict transcriptional regulation, orchestrated predominantly by factors suppressing expression of the *Air-1* gene, which encodes the MHC class II trans-activator (CIITA) [14]. This ensures that MHC II display is normally limited to professional antigen-presenting cells (APCs) such as B cells, dendritic cells and macrophages. However, some non-APC tumor cells can express MHC class II molecules under certain experimental conditions (reviewed in [15]). For example, the B16 melanoma cell line has no constitutive MHC II expression, but up-regulate MHC II expression in the presence of IFN-γ [1, 16]. It has further been shown that B16 cells express MHC II *in vivo*, and can be directly recognized and eliminated by CD4^+^ T cells specific for the tumor-specific antigen Trp-1 [1, 16]. These findings raise the relevant question of whether inducible, tumor cell-intrinsic MHC II display may explain CD4^+^ T cell-mediated killing of seemingly MHC II^NEG^ tumor cells.

*In vitro* cultured or *ex vivo*-isolated MOPC315 myeloma tumor cells do not show detectable MHC class II expression, as assayed by flow cytometry or functional assays [8, 13, 17]. Nevertheless, MHC II (I-E^d^)-restricted Ig V-region (Idiotype, Id)-specific CD4^+^ T cells of T-cell-receptor-transgenic (TCR-Tg) mice reject MOPC315 cells [18, 19]. These mice harbor CD4^+^ T cells specific for a mutated IgA λ2^315^ variable region epitope (aa91–101), presented in the context of MHC II I-E^d^. In this model, elimination of tumor cells appears to occur through an indirect mechanism that involves secretion of myeloma protein [20, 21]. Secreted antigen is taken up, processed and presented by tumor infiltrating macrophages [13]. Recognition by tumor infiltrating Th1 cells results in an IFN-γ-dependent induction of a cytotoxic M1 macrophage phenotype, resulting in tumor killing [13, 22]. However, it may be argued that despite the lack of constitutive MHC II expression, MOPC315 cells may start to express MHC class II under certain *in vivo* conditions, as observed for the B16 melanoma [1, 16]. This argument is particularly relevant for myeloma cells, which belong to the B cell lineage, members of which express MHC class II molecules at certain stages of their differentiation.

*In vitro* analyses reveal that MOPC315 cells produce factors that prevent expression of CIITA. Nonetheless, MHC II expression can be restored by epigenetic modifications. Therefore, to conclusively resolve the issue of the role of MHC class II display on tumor cells, we generated MOPC315 cells deficient in MHC class II by ablation of the *H2eb1* gene, encoding the b-chain of the relevant MHC II molecule (I-E^d^). Our results show that Id-specific CD4^+^ T cells were able to reject MHC II deficient MOPC315 cells, conclusively demonstrating that CD4^+^ T cells can kill MHC II^NEG^ tumor cells.

## RESULTS

### MOPC315 myeloma cells lack constitutive or IFN-γ-inducible MHC class II expression

**Figure 1:**
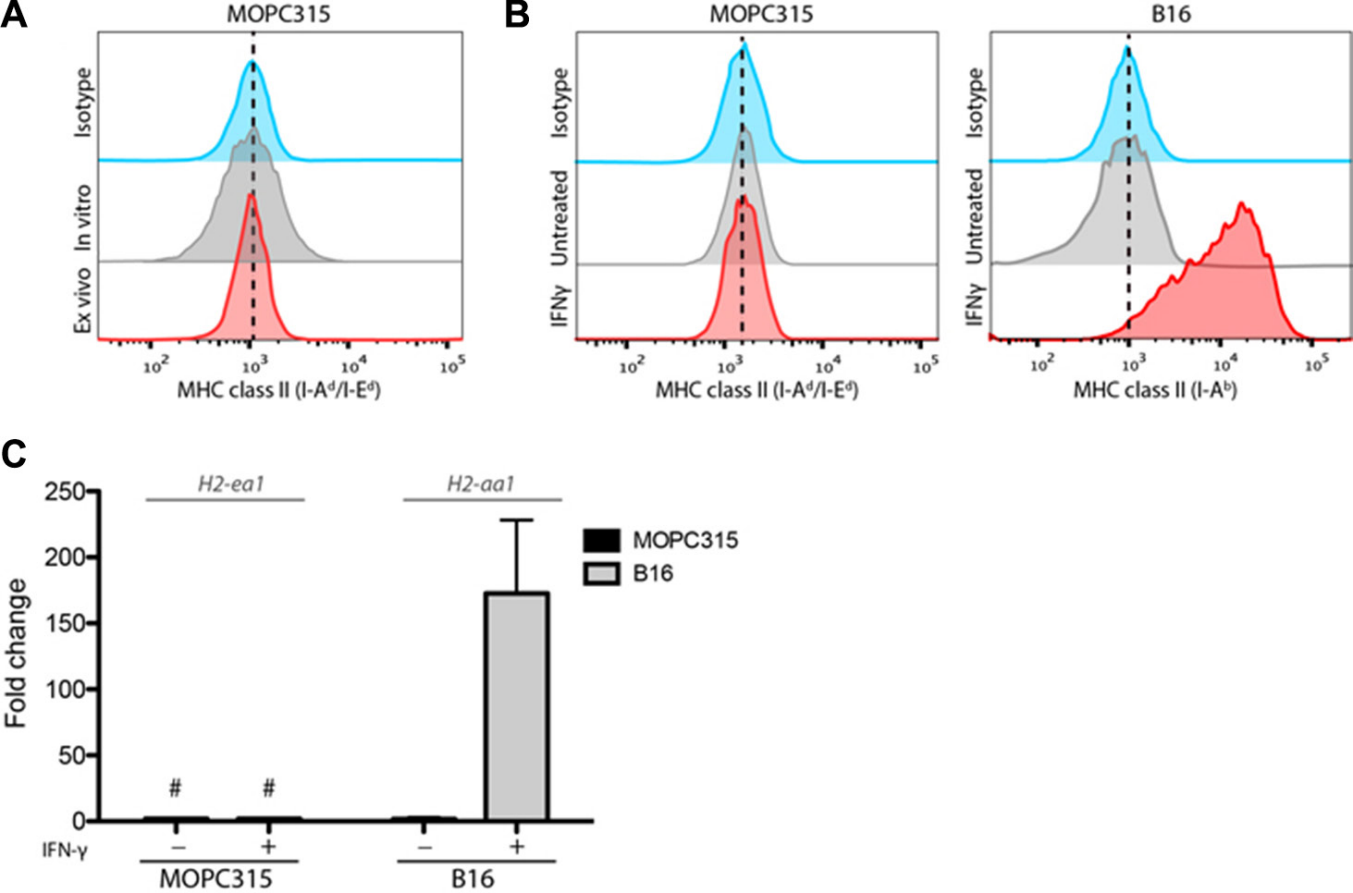
MOPC315 cells do not express MHC class II (**A**) Representative flow cytometry staining for MHC class II (I-A^d^/E^d^) on MOPC315 cells cultured *in vitro* or stained directly after isolation (*ex vivo*) from s.c. matrigel tumors on d+12 after challenge in TCR-Tg mice. (**B**) Flow cytometry histograms showing MHC class II expression in MOPC315(I-A^d^/E^d^) and B16 (I-A^b^) cells after 24 h incubation in the presence or absence of IFN-γ (100 units/ml). (**C**) mRNA expression of the gene encoding the relevant MHC class II alpha chain in MOPC315 (I-E^d^; H2-iae) and B16 (I-A^b^;H2-iaa) cells treated as in (B). Data shows mean expression + SD relative to untreated controls (fold change). # − no detectable signal. (*n* = 4 per treatment group).

In line with previous reports [8, 13, 17], both *in vitro*-cultured MOPC315 cells and tumor cells analyzed directly after *ex vivo* isolation from subcutaneous or bone marrow tumor foci showed no detectable expression of MHC class II by flow cytometry (Figure 1A). Tumor cells also failed to support proliferation of Id-specific CD4^+^ T cells in the presence of synthetic Id peptide (data not shown).

Interferon γ (IFN-γ) signaling is considered an important part of Th1 responses against tumors. IFN-γ is a well-known inducer of MHC class II expression in some tumor cell lines, including the C57Bl6-derived (H2^b^ haplotype) B16 melanoma [16].

In contrast to B16, MOPC315 cells (BALB/c-derived, H2^d^ haplotype) failed to express MHC class II after 24 h incubation with high dosages of IFN-γ (Figure 1B). Long-term exposure to IFN-γ (100–1000ng/mL) for up to 72 hours did not result in expression of MHC class II (data not shown). Similarly, IFN-γ stimulation had no effect on mRNA expression levels of the *H2-ea1* gene, encoding the MHC II I-E^d^ alpha chain (Figure 1C).

### MOPC315 cells express a dominant suppressor of the Air-1 gene, susceptible to modulation by epigenetic modification

In order to further define the mechanistic basis of the lack of MHC II expression, we performed fusion experiments using either the BALB/c-derived A20 lymphoma cell line, which constitutively expresses MHC II (I-A^d^/I-E^d^), or the C57BL/6-derived B16 melanoma (I-A^b^), which expresses MHC II upon IFN-γ stimulation (cfr. Figure 1B).

**Figure 2:**
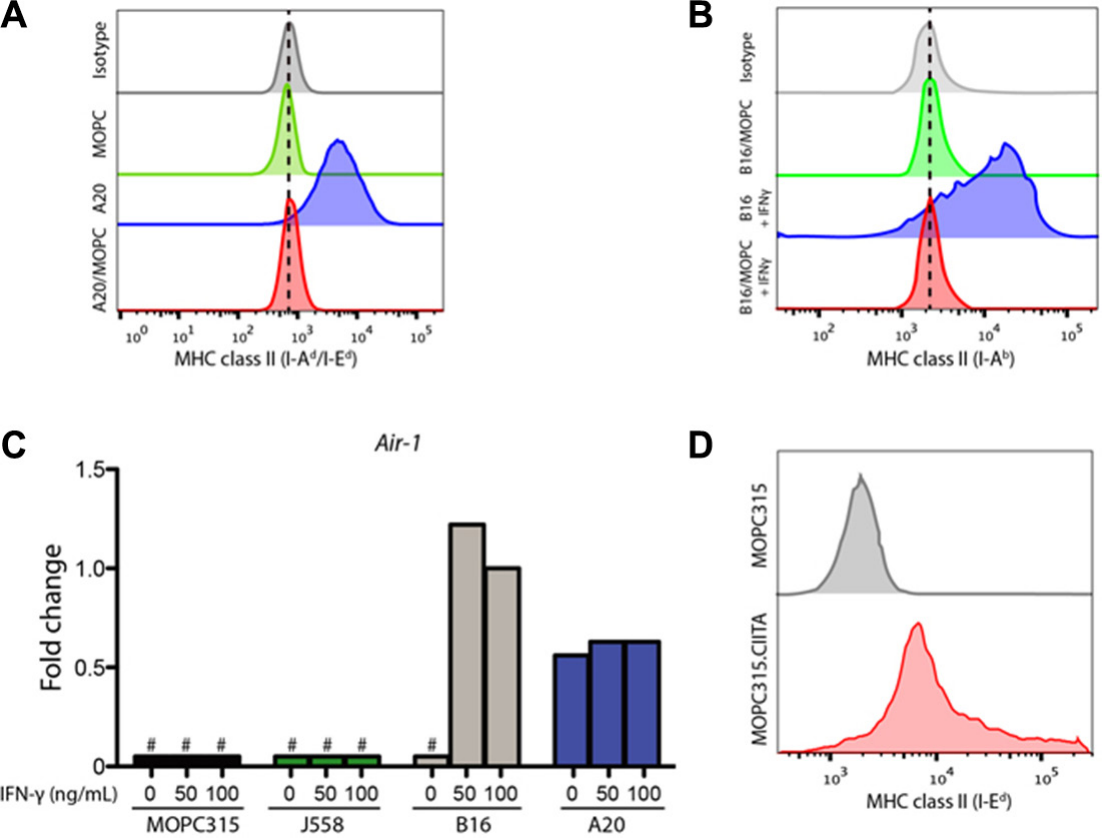
MOPC315 cells contain dominantly suppressive factors preventing MHC II expression (**A**) Flow cytometry data showing surface MHC class II expression (I-A^d^/E^d^) on A20, MOPC315 and A20/MOPC315 fusion cells. (**B**) Surface MHC class II (I-A^b^) expression in B16 and B16/MOPC315 fusion cells cultured for 24 h in the presence or absence of 100U/mL IFN-γ. (**C**) mRNA expression of the *Air-1* gene in MOPC315, J558, B16 and A20 cells treated with IFN-γ at the indicated concentrations for 24 h. Results relative to B16 cells exposed to IFN-γ (fold change). Data represents the mean of 4 replicates per treatment group. # − no detectable expression. (**D**) Surface staining of MHC class II (I-E^d^) in wild type MOPC315 cells compared to a transfectant expressing human CIITA (MOPC315.CIITA).

Cloned MOPC315/A20 fusion cells showed no detectable MHC II expression (Figure 2A). Similarly MOPC315/B16 fusions lacked detectable expression of I-A^d^, I-E^d^ and I-A^b^ after IFN-γ stimulation (Figure 2B). These results indicate that MOPC315 cells contain factors that dominantly suppresses constitutive, as well as IFN-γ-induced, MHC II expression.

Expression of MHC class II gene requires the presence of CIITA, encoded by the *Air-1* gene [23]. Real-time PCR demonstrated a lack of detectable *Air-1* expression in MOPC315, J558 or B16 cells, while it was present in the A20 lymphoma (Figure 2C). *Air-1* was significantly induced after IFN-γ stimulation of B16 cells, but remained undetectable after IFN-γ stimulation in MOPC315 and J558 myeloma cells (Figure 2C). We therefore hypothesized that the lack of MHC II expression on MOPC315 was due to the absence of CIITA expression. Accordingly, we overexpressed human CIITA in MOPC315 cells, and observed restoration of MHC II expression (Figure 2D).

**Figure 3:**
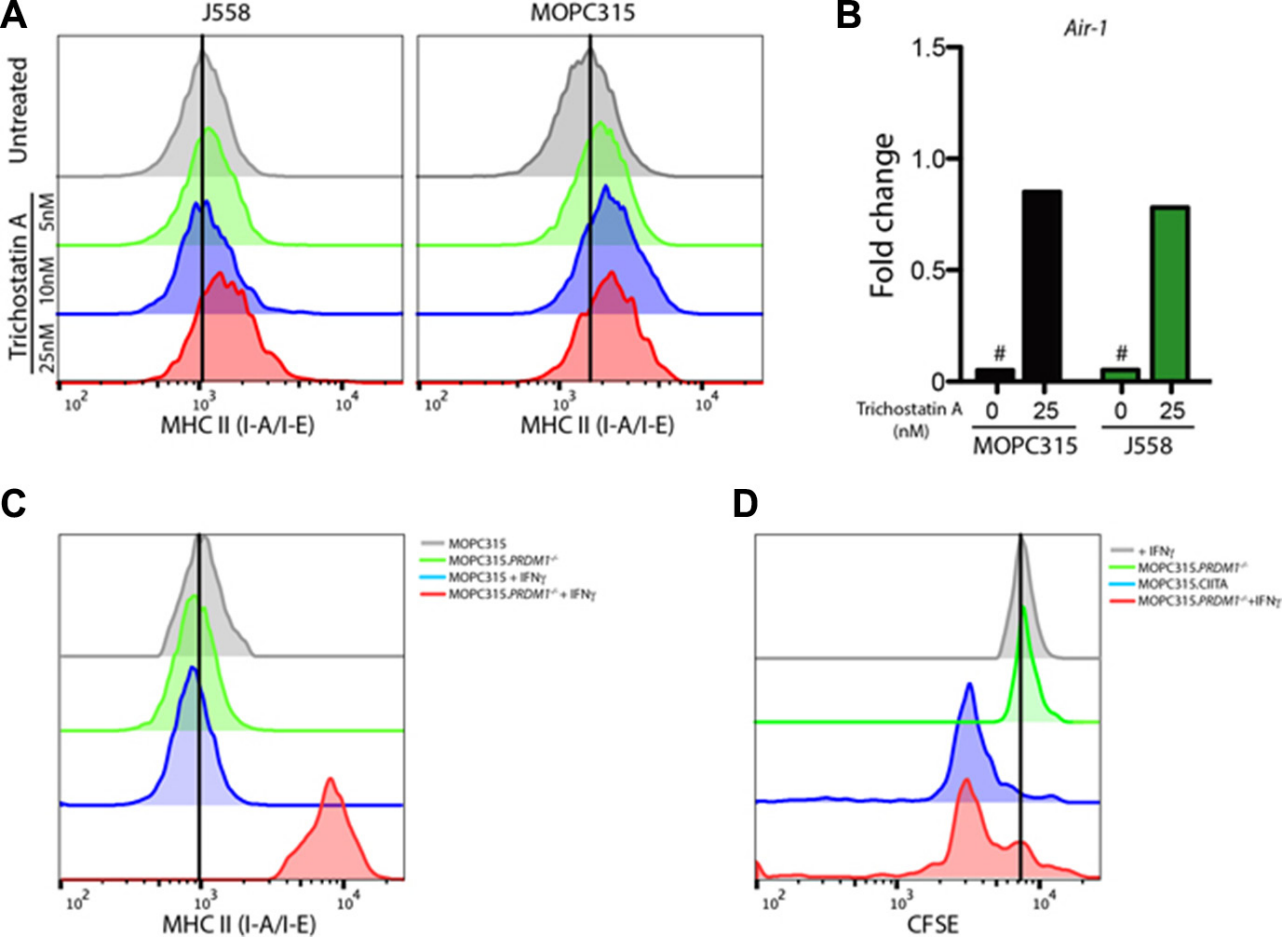
HDAC inhibitor treatment restores MHC class II expression in myeloma cells (**A**) Flow cytometry measurements of surface MHC class II expression ((I-A^d^/E^d^, MFI) in J558 and MOPC315 myeloma cells after 24 h incubation with the HDAC inhibitor Trichostatin A at the indicated concentrations. (**B**) mRNA expression of the *Air-1* gene in MOPC315 or J558 cells treated as in (A), plotted relative to expression in J558 cells treated with 10 nM Trichostatin A (fold change). Data represent mean of 4 replicates per group. # − no detectable expression. (**C**) Representative flow cytometry quantitation of MHC class II expression (MFI) on wild type MOPC315 and MOPC315. *Prdm1^−/−^* cells incubated in the presence or absence of IFN-γ (100 units/mL) for 24 h. (**D**) Flow cytometry analysis of CFSE-labeled naïve Id-specific CD4^+^ T cells (1 × 10^4^) co-incubated with the indicated MOPC315 variant (3 × 10^4^) for 72 h. Markers indicate percentage of cells showing CFSE dilution, indicative of T cell proliferation. Results are representative of two independent experiments.

A previous report has shown that MHC II expression in another murine myeloma cell line, J558, was suppressed by epigenetic mechanisms, and could be reversed by treatment with the histone deacetylase inhibitor Trichostatin A (TSA) [24]. A moderate surface expression of MHC class II was detectable in MOPC315 cells after 24 h treatment with TSA, comparable to that of J558 cells (Figure 3A), indicating a similar mode of epigenetic silencing. In agreement with these findings, TSA stimulation induced Air-1 transcription in both cell lines (Figure 3B). TSA-induced MHC II expression remained high after several weeks of *in vitro* culture (*data not shown*). We thus conclude that epigenetic modifications may override the suppression of MHC class II seen in myeloma cells, including MOPC315.

### BLIMP-1 expression restricts IFN-γ-inducible MHC II expression in myeloma cells

It has been previously found that B lymphocyte-induced maturation protein I (BLIMP-I) underlies the lack of IFN-γ-inducible MHC II expression in plasma cells [25]. We therefore hypothesized that constitutive expression of BLIMP-I may explain the suppression of MHC II display in myeloma cells. Ablation of the BLIMP-I-encoding *Prdm1* gene using CRISPR/Cas9 (Figure S1) restored sensitivity of MHC II expression to IFN-γ stimulation (Figure 3C), resulting in functional display of an endogenous Id to Id-specific CD4^+^ T cells *in vitro* (Figure 3D). This identifies BLIMP-I-mediated repression of CIITA expression as a key contributor to the MHC II negativity of MOPC315 cells.

### MHC II expression on tumor cells is not required for *in vivo* CD4^+^ T cell immunoprotection

**Figure 4:**
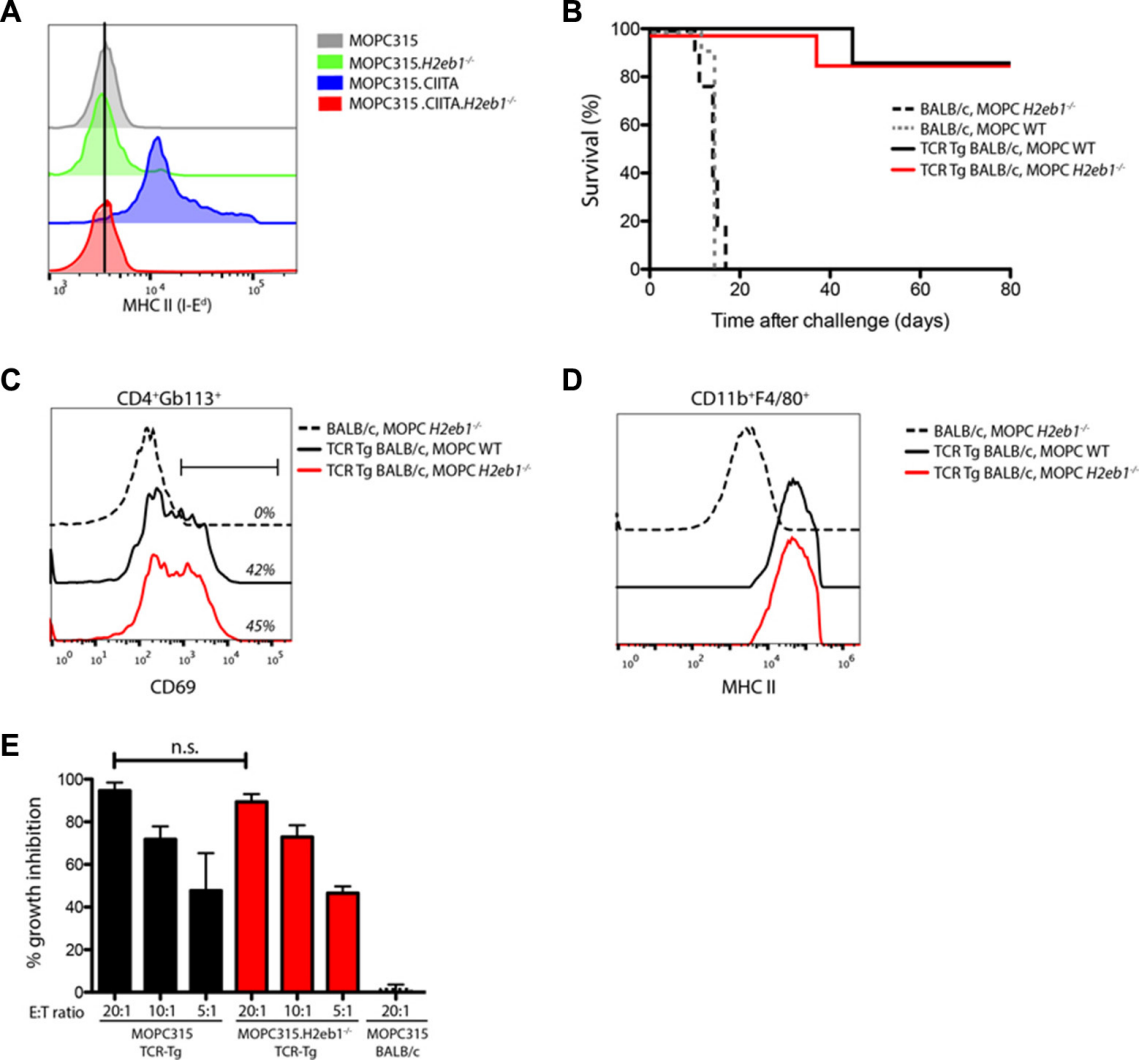
MHC II expression on tumor cells is not required for CD4+ T cell immunoprotection (**A**) Surface MHC II (I-E^d^) staining in wild type MOPC315 and MOPC315.*H2eb1*^−/−^ clones transfected with human CIITA. (**B**) Survival curves of TCR-Tg BALB/c and BALB/c mice challenged s.c. with 1.6 × 10^5^ wild type (WT) or MHC II(I-E^d^)-deficient (*H2eb1*^−/−^) MOPC315 cells. *n* = 8–12 per group. (**C**) Representative flow cytometry staining for the activation marker CD69 on Id-specific (GB113^+^) CD4^+^ T cells from draining lymph nodes isolated on day +10 after challenge with WT or MHC II-deficient MOPC315 cells as described in (B). (**D**) Representative flow cytometry staining for MHC class II (I-A^d^/E^d^) on tumor-infiltrating CD11b^+^F4/80^+^ cells isolated from tumor-containing matrigels on day +10 after challenge with WT or MHC II-deficient MOPC315 cells as described in (B). (**E**) *In vitro* co-culture assays demonstrating growth inhibitory effects of CD11b^+^ cells isolated from Matrigel tumors on day +10 after challenge with 1.6 × 10^5^ wild-type (black bars) or MHC II-deficient (*H2eb1*^−/−^, red bars) MOPC315 cells in respectively TCR-Tg and BALB/c mice. Isolated CD11b^+^ cells were mixed with wild-type MOPC315 cells at the indicated ratios and co-cultured for 48 h. Growth inhibitory effect is expressed as % of tumor cells cultured alone. Data is represented as mean + SD (*n* = 4–8 per treatment group). n.s. − not significant.

Despite the lack of detectable MHC class II expression in wild type MOPC315 cells, our results indicated that suppression of MHC II may potentially be reversed under certain circumstances. To definitely exclude the possibility of inducible MHC II I-E^d^ expression on tumor cells, we generated an MHC II-deficient variant (MOPC315.*H2eb1^−/−^*) by ablating the *H2eb1* (encoding the b-chain of the relevant MHC II molecule I-E^d^) locus using CRISPR/Cas9. Successful knockout was verified by lack of expression of MHC II after overexpression of CIITA in the individual clones (Figure 4A) and by gene sequencing (*data not shown*). These results indicate that all *H2eb1* genes had been functionally disrupted.

We next proceeded with s.c. tumor challenge experiments using the MOPC315.*H2eb1^−/−^* cell line. BALB/c mice injected with the MOPC315.*H2eb1^−/−^* cell line developed tumors with the same kinetics as mice injected with the wild type (WT) MOPC315 cell line (Figure 4B). In contrast, Id-specific TCR-Tg BALB/c mice were completely protected against challenge with either MOPC315.*H2eb1^−/−^* or WT MOPC315 cells (Figure 4B). Analyses of draining lymph nodes of TCR-Tg mice revealed that CD4^+^ T cell activation induced by MOPC315.*H2eb1^−/−^* was equal to that observed after challenge with wild type MOPC315 cells (Figure 4C). Similarly, macrophage activation obtained with MOPC315.*H2eb1^−/−^* in TCR-Tg mice was comparable to that observed with WT MOPC315, as measured by increased MHC II expression levels (Figure 4D) and *ex vivo* cytotoxicity (Figure 4E). In summary, these findings conclusively demonstrate that Id-specific CD4^+^ T cells induce protective anti-tumor immune responses via indirect recognition of the tumor antigen. Such immune responses are therefore not dependent on MHC II expression on the tumor cells themselves.

## DISCUSSION

Our results demonstrate that MHC class II molecule expression is not required for CD4^+^ T cell-mediated rejection. This raises the question of whether tumor-cell-intrinsic MHC class II expression is an absolute requirement for tumor rejection, even in settings where the majority of tumor cells do express MHC class II. Although several authors have noted that MHC II expression on tumor cells can be induced *in vitro* [23] and *in vivo* [1, 16], the extent and functional relevance of such changes remains unclear. In the case of B16 melanoma, optimal outcome required sub-lethal irradiation and anti-CTLA-4 mAb injections in addition to adoptive T cell transfer (ACT) [1]. It is possible that combinatorial treatment regimens may induce changes that facilitate or enhance MHC II display on tumor cells, thereby enabling direct CD4^+^ T cell cytotoxicity. The finding that epigenetic modifications, e.g. using a HDAC inhibitor, may result in MHC II display, further underscore the potential for therapy-induced enhancement of ectopic antigen display on tumor cells. It has been shown that irradiation, as well as chemotherapy, may trigger MHC class I expression on tumor cells [26], and a similar impact on MHC II expression could be envisioned.

Recent data from our lab suggests that MHC II-deficient myeloma cells can be eliminated by a combinatorial regimen of CD4^+^ ACT and sub-lethal irradiation in the setting of established, disseminated myeloma within the bone marrow [2], although the effector cells in this setting have not been identified. In conclusion, the present results unequivocally demonstrate the ability of CD4^+^ T cells specific for a secreted tumor antigen to eliminate MHC II-negative tumor cells. Ablation of the MHC II (I-E^d^) beta chain did not affect T cell activation in draining lymph nodes, or the induction of cytotoxicity in tumor-associated macrophages. Hence, neither the priming nor the effector phase of the CD4^+^ T cell response was affected by the absence of tumor cell-intrinsic MHC II display. These results provide direct confirmation of the hypothesis that indirect display of tumor-specific antigens on tumor-infiltrating APCs is sufficient for CD4^+^ T cells-mediated tumor rejection [10, 13, 20]. Given the key role of CD4^+^ T cells as orchestrators of adaptive immune responses, there is increasing interest in the use of this T cell subset in immunotherapy against cancer. As illustrated by the present findings, CD4^+^ T cells may trigger cell signaling programs and release of cytokines and other factors that alter the tumor microenvironment. The present findings show that such indirect effects may confer therapeutic benefit *in vivo*. These findings highlight a largely overlooked mode of tumor immunosurveillance, and warrant further evaluation of the relative importance of direct versus indirect antigen display in CD4^+^ T cell responses against cancer. This insight may guide the selection of antigen targets and choice of conditioning regimen in immunotherapeutic interventions utilizing tumor-specific CD4^+^ T cells.

In summary, our results provide evidence that indirect display of a secreted tumor antigen on infiltrating macrophages is sufficient for killing of MHCII^NEG^ tumor cells by CD4^+^ T cells.

## MATERIALS AND METHODS

### Cells, cell lines, and cell fusion

The BALB/c-derived A20 lymphoma and MOPC315 plasmacytoma cell lines, and the C57Bl/6-derived B16 melanoma were obtained from the American Type Culture Collection (ATCC, Manassas, VA). The MOPC315.4 clone used in these and our previous experiments (hereafter referred to as MOPC315) was derived by *in vivo* passaging to ensure consistent tumor take [8]. mCherry- and EGFP-expressing variants were generated by lentiviral transduction, as previously described [21]. All cells were maintained in RPMI 1640 GlutaMAX (Invitrogen, Carlsbad, CA) supplemented with 10% FCS (Greiner Bio-one GmbH, Frickenhausen, Germany) and penicillin/streptomycin.

Cell fusion was performed according to a previously published protocol [27], with some modifications. Briefly, 1 × 10^7^ of each of EGFP- or mCherry-expressing variants of the cell types to be fused were mixed, washed extensively and resuspended in 1 mL 50%(w/v) polyethylene glycol (PEG1500; Sigma-Aldrich) in 75 mM Hepes (pH 8.0). The cells were then incubated for 1 minute in a 37C water bath, and RPMI1640 medium added drop-wise. Cells were maintained in complete medium, and successfully fused cells were isolated by FACS sorting (FACS Aria II; BD Biosciences) of single cells with dual expression of EGFP and mCherry after two days in culture. Cell fusion was verified by fluorescence microscopy immediately after sorting and in the course of subsequent culture.

### Antibodies and flow cytometry

Non-specific binding was blocked by incubation with heat-inactivated (56°C, 30 min) 30% normal rat serum in PBS and 100 μg ml^−1^ anti-FcγRII/III monoclonal antibodies (mAb; clone 2.4G2). Cells were stained for 15 min on ice with specific mAbs in PBS supplemented with 0.5% bovine serum albumin (Biotest). The following commercially available mAbs were used, conjugated with either FITC, PE, or Pe-Cy7: CD3 (17A2), CD4 (GK1.5), MHC class II I-E^d^ (14-4-4S) (BD Biosciences), MHC class II I-A/E (M5.114.15.2) (Biologend), MHC class II I-A^b^ (AF6-120.1). The following mAbs were affinity-purified and, if needed, biotinylated in our laboratory: anti-Id-specific-TCR-clonotype (GB113), anti-FcγRII/III (2.4G2; ATCC). Biotinylated mAbs were detected with streptavidin conjugated to peridinin chlorophyll protein (BD Biosciences). Cells were analyzed on a Fortessa instrument with FACSDiva software (BD Biosciences).

### Mice, tumor challenge and tissue preparation

BALB/c mice were purchased from Taconic (Taconic farms, Rye, Denmark). Homozygous Id-specific T cell receptor transgenic (TCR-Tg) BALB/c mice were generated and maintained as previously described [19]. These mice harbor CD4^+^ T cells specific for a mutated IgA λ2^315^ variable region epitope (aa91-101) presented in the context of MHC II I-E^d^. 4-6 week old mice were injected subcutaneously in one flank with 1.6×10^5^ tumor cells suspended either in 100 μL phosphate-buffered saline (PBS), or in 200 μL growth factor-reduced Matrigel (BD Biosciences, San Diego, CA). Tumor growth was followed by palpation and electronic caliper measurements. Mice were euthanized when tumor diameter exceeded 15 mm. Tumor cells were recovered from matrigel as previously described [28]. Briefly, Matrigel plugs were dissociated by 30 min incubation at 37°C in RPMI1640 medium containing collagenase/DNase. Cells were recovered by centrifugation at 800× g and washed using PBS. Tumor-infiltrating macrophages were enriched using CD11b MicroBeads beads (Miltenyi Biotech, Bergisch Gladbach, Germany), according to the manufacturers instructions. Id-specific T cells were isolated from spleens and lymph nodes from TCR-Tg BALB/c mice using MACS beads (CD4^+^ T Cell Isolation Kit II; Miltenyi).

### Transfection, transduction and CRISPR/Cas9 gene ablation

Generation of mCherry- and GFP-expressing MOPC315, B16 and A20 cells was performed by lentiviral transduction, as previously described [22].

An expression vector encoding the human CIITA protein was a kind gift from dr. Matja Peterlin, deposited through Addgene (#14650). Stable transfectants were generated by electroporation using the Amaxa Nucleofector II (Lonza, Basel, Switzerland) with subsequent selection using medium containing puromycin.

CRISPR/Cas9 experiments were performed according to published guidelines [29]. The vector pSpCas9(BB)-2A-GFP (PX458) was a gift from Feng Zhang (Addgene plasmid # 48138). RNA guide sequences were generated using the online service CHOPCHOP [30]. The following guide sequences were utilized: H2eb#1: 5′-GCCACACCATGCTGCAGGAG-3′, H2eb#2: 5′-GTATTCCAAAAACCATGCTG-3′, *Prdm1#*1: 5′-GG AGCCGGAGCTAGACTTGG-3′. Target cells were electroporated using the Amaxa Nucleofector II (Lonza; Program U16), and GFP-positive cells were single-cell sorted 24 h later using a FACSAria II (BD Biosciences). The relevant gene segment was isolated from individual clones by standard TOPO cloning and sequenced. Clones showing non-sense mutations were further evaluated by flow cytometry and T cell proliferation assay after overexpression of human CIITA (see results section) to exclude MHC II expression.

### Real-time PCR

Total RNA was extracted from cultured cells using the RNeasy Mini kit (Qiagen, Hilden, Germany), and cDNA generated using the 1st Strand cDNA Synthesis Kit (Life Technologies, Grand Island, NY) with oligo-(dT) primers. RT-PCR was performed using TaqMan Fast Universal PCR Master Mix on an ABI-Prism 7000 Sequence Detection system (Applied Biosystems, Foster City, CA) using pre-designed FAM/TAMRA primer/probe-sets (IDT Technologies, Coralville, IO). Relative expression (fold change) was calculated by the ddCt method with normalization against TATA box binding protein (Tbp), with reference group as specified in figure legends.

### *In vitro* growth inhibition assay

Tumor-infiltrating CD11b^+^ cells were purified at day +11 after s.c. injection by positive selection using CD11b MicroBeads (Miltenyi Biotec GmbH, Gladbach, Germany) according to the manufacturer's instructions. Macrophages/CD11b^+^ cells were irradiated (2000 rad) and added at various effector/target ratios to MOPC315 tumor cells. Cells were pulsed with ^3^H-thymidine after 48 h and collected 12 h later on a TopCount NXT microplate counter (PerkinElmer, Shelton, CT).

### Statistical analysis

The Mann–Whitney *U* test was used for statistical analysis unless stated otherwise. For tumor challenge experiments, differences in survival were analyzed using the log-rank test. Statistical analysis was performed using Prism 5.0 software (GraphPad Software, La Jolla, CA). *p* < 0,05 was considered statistically significant.

## SUPPLEMENTARY MATERIAL FIGURE


