# Supplementary material for: Tumor-specific CD4+ T cells eradicate myeloma cells genetically deficient in MHC class II display
Source: Oncotarget. 2016 Sep 10;7(41):67175–82. doi: 10.18632/oncotarget.11946 (PMC5341866; doi:10.18632/oncotarget.11946)
Supplement: Supplementary file 1 [file oncotarget-07-67175-s001.pdf]

Tumor-specific CD4⁺ T cells eradicate myeloma cells genetically deficient in MHC class II display

Supplementary Materials

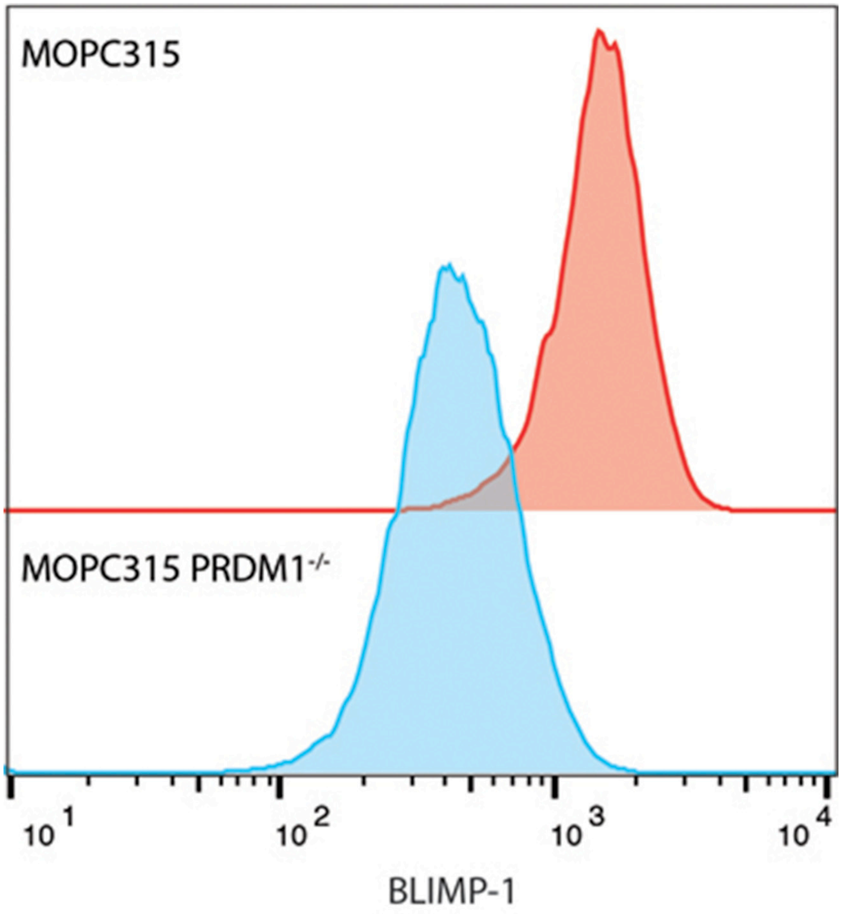

Supplementary Figure S1: Intracellular BLIMP1 protein expresion is effectively ablated by the distruption of the *PRDM1* gene (MOPC315 PRDM1$^{-/-}$).
